# A post-marketing surveillance study of a human rabies vaccine in China (2020–2024)

**DOI:** 10.3389/fpubh.2026.1741521

**Published:** 2026-07-14

**Authors:** Zhijie Cui, Lianfu Wang, Zhiyuan Ran, Yuan Liu, Yawei Gan, Wuyang Zhu

**Affiliations:** 1 Aerospace Center Hospital, Beijing, China; 2 Beijing Taikang Yanyuan Rehabilitation Hospital, Beijing, China; 3 Hu'an Biotechnology (Beijing) Co., Ltd, Beijing, China; 4 Department of Rabies, National Institute for Viral Disease Control and Prevention, Chinese Center for Disease Control and Prevention, Beijing, China

**Keywords:** adverse events, post-marketing surveillance, rabies vaccine, safety, Vero cell, Yisheng Jun’an®

## Abstract

**Background and objective:**

To evaluate the safety profile of Yisheng Jun’an® freeze-dried human rabies vaccine (Vero cell) through post-marketing surveillance in China (2020–2024), focusing on adverse reaction patterns and influencing factors.

**Methods:**

A retrospective observational study analyzed 2,419 adverse events from the national Direct Adverse Event Reporting System (DAERS) and manufacturer-collected data. Adverse reactions were categorized by type, severity, and subgroup (age, gender, dose number, etc.) using the Medical Dictionary for Regulatory Activities (MedDRA) for standardized coding. Analysis of incidence rates with 95% confidence intervals, use of the chi-square test to assess associations, and cross-subgroup comparisons were conducted to elucidate the safety profile of the vaccine.

**Results:**

In total, 2,419 AEs were reported, yielding an overall incidence of 7.406 per 100,000 doses (95% CI 7.11–7.71). Systemic reactions comprised 83.38% and local reactions 16.62%. Seventeen serious adverse events (0.70%) occurred; no deaths were reported and all recovered. The most common categories were general disorders/administration-site conditions (83.38%) and immune system disorders (5.70%). AEs were most frequent after the first dose (85.49%) and declined markedly with subsequent doses. Systemic reactions were more common in children (0–10 years: 37.99%).

**Conclusion:**

Yisheng Jun’an® rabies vaccine exhibits a favorable safety profile with mild-to-moderate reactions, supporting its use in post-exposure prophylaxis.

## Introduction

Rabies is an acute and almost invariably fatal zoonotic disease of the central nervous system caused by the rabies virus (1). Following exposure, the virus replicates locally in muscle tissue and subsequently invades peripheral nerves, spreading rapidly to the spinal cord and brain, where it induces acute encephalitis or meningoencephalitis (2, 3). Clinical manifestations include hydrophobia, aerophobia, pharyngeal muscle spasms, and progressive paralysis (4, 5). Once clinical symptoms occur, the case fatality rate approaches 100% (6). Although rabies incidence in China has declined with improved public awareness, standardized post-exposure prophylaxis (PEP), and wider access to vaccines, the disease still results in more than a hundred human deaths annually (6, 7). In the absence of effective therapeutic drugs, timely wound management, active immunization with rabies vaccines, and where indicated, administration of rabies immunoglobulin remain the most effective measures for prevention and control (8, 9).

Among the four types of cell-culture based rabies vaccines currently approved in China, the purified Vero cell rabies vaccine has become widely used due to its favorable safety profile, robust immunogenicity, and cost-effectiveness (10). Yisheng Jun’an® (YSJA), a freeze-dried purified Vero cell rabies vaccine developed by Yisheng Bio-Pharma, has been marketed since 2003 and has been extensively applied in nationwide PEP programs. The vaccine is produced by culturing the CTN-1 strain in Vero cells. It is formulated without an aluminum adjuvant and lyophilized, resulting in a stable product with a shelf life of up to 36 months. Previous clinical studies have demonstrated that YSJA has a generally favorable safety profile, with adverse reactions mostly limited to mild, transient local or systemic symptoms (11). Nevertheless, considering the large and diverse population exposed to rabies vaccination, systematic evaluation of adverse event profiles remains essential. The present study therefore retrospectively analyzes reported adverse reactions following YSJA vaccination from 2020 to 2024, aiming to clarify incidence patterns, risk factors, and implications for optimizing vaccine safety surveillance and clinical practice.

## Methods

### Study design and data sources

This was a retrospective, observational post-marketing surveillance study without a control group, conducted in medical institutions and Centers for Disease Control and Prevention (CDCs) across mainland China. We analyzed case data of abnormal reactions following the vaccination of Yisheng Jun’an® freeze - dried human rabies vaccine from October 2020 to November 2024. Data for this retrospective study were derived from two parts: one was all individual case information related to Yisheng Jun’an® rabies vaccine reported through DAERS; the other was adverse reaction data actively solicited by the enterprise. All reported cases were graded for severity in accordance with the Chinese National Medical Products Administration’s 2019 “Guidelines for Grading Adverse Events in Clinical Trials of Preventive Vaccines,” with relevant data and records undergoing medical review.

The study period was defined as October 2020 onward because, in December 2019, the Chinese National Medical Products Administration (NMPA) issued a new version of the Guidelines for Grading Adverse Events in Clinical Trials of Preventive Vaccines, which provided more standardized and detailed criteria for severity grading of adverse events following immunization. To ensure consistency and comparability of adverse event severity assessment across all reported cases, we included only data collected after the new guideline had been fully implemented in the national surveillance system (DAERS) and by the manufacturer. Data prior to October 2020 were based on older grading standards and were therefore not directly comparable.

All symptom descriptions of adverse events were medically coded and entered by trained personnel using the clinically validated international standardized medical terminology, the Medical Dictionary for Regulatory Activities (MedDRA, Version 25.0), grouped by System Organ Class (SOC). Preferred Terms (PTs) were assigned to describe specific adverse events, while High-Level Group Terms (HLGTs) and System Organ Class (SOC) codes were used for categorical classification and analysis. Exposure to Yisheng Jun’an® freeze-dried human rabies vaccine (Vero cell) was identified in the Direct Adverse Event Reporting System (DAERS) and manufacturer-collected data using the specific product name Yisheng Jun’an® and its national drug approval number (S20040007). The entire study adhered to the Declaration of Helsinki and Good Clinical Practice (GCP) requirements.

### Vaccine and immunization schedule

The vaccine involved in this post - marketing surveillance study was Yisheng Jun’an® freeze-dried human rabies vaccine (Vero cell) produced by Liaoning Yisheng Bio-Pharmaceutical Co., Ltd. The vaccine was prepared by inoculating the fixed rabies virus strain CTN-1 into Vero cells, followed by cultivation, harvest, concentration, virus inactivation, and purification.

For post-exposure prophylaxis, the vaccine was administered in a five-dose schedule. A total of 5 doses were inoculated on days 0 (the day of exposure), 3, 7, 14, and 28 after exposure. When using the freeze-dried vaccine, it was reconstituted with the diluent to 0.5 mL for inoculation. For children over 2 years old and adults, the vaccine was inoculated into the deltoid muscle of the upper arm, while for children under 2 years old, it was inoculated into the anterolateral muscle of the thigh.

### Indicators

#### General information and other Clinicopathological characteristics

Variables included age groups (0–10 years old, 11–20 years old, 21–40 years old, 41–60 years old, >60 years old), gender, vaccination year (2020, 2021, 2022, 2023, 2024), dose number at which the adverse reaction occurred (1st, 2nd, 3rd, 4th, 5th dose), reporting source (ADR, CDC, others), System Organ Class (SOC), number of local adverse events, and total number of systemic adverse events.

#### Reactogenicity and allergic reactions

Reactogenicity was defined as common local and systemic reactions, including redness, swelling, pain, fever, arthritis, nephritis, chills, nausea, vomiting, hypotension, and organ dysfunction. Allergic reactions included rash, pruritus, angioedema, respiratory symptoms (chest tightness, shortness of breath, dyspnea, asthma attack), and others (palpitations, syncope, dizziness, fatigue, convulsions, shock). The number of cases, frequency of each symptom were recorded. All adverse events observed within 30 min after each vaccination and those occurring within 7 days after vaccination were considered vaccine - related. The causal relationship of all other adverse events was evaluated based on clinical judgment.

#### Serious adverse events

All serious adverse events that occurred within 6 months after the completion of the full-course vaccination and led to hospitalization, significant disability, or death were summarized on a case-by-case basis.

### Statistical methods

During data cleaning and collation, a double-checking mechanism was used to clean the raw data to ensure data integrity and accuracy. A data dictionary was established to standardize and code variables using MedDRA, including age, gender, geographical distribution, classification of adverse reactions, and severity. Continuous variables were presented as median (interquartile range). Categorical variables were presented as frequency (n) and percentage (%).

The incidence rates of local and systemic reactions (number of events/total number of vaccination doses × 100,000) and their 95% confidence intervals (95% CI) were calculated using the Clopper-Pearson method. Chi-square test or Fisher’s exact test was used to analyze differences among categorical variables (such as gender, age group, and occurrence of adverse reactions). Data analysis was performed using SPSS 25.0 software. The significance level was set at α = 0.05 for two-tailed tests, and p < 0.05 was considered statistically significant.

## Results

### Incidence of reported adverse events following immunization (AEFI)

#### General findings

A total of 2,419 adverse events were reported. Local adverse reactions accounted for 402 cases (16.62%), and systemic adverse reactions were 2,017 cases (83.38%), with an overall incidence of 7.406 per 100,000 doses (95% CI: 7.11–7.71). A total of 17 serious adverse events (SAEs) were reported, accounting for 0.70% of all adverse events, with no deaths. All cases have fully recovered.

#### Patterns of distribution

**Figure 1:**
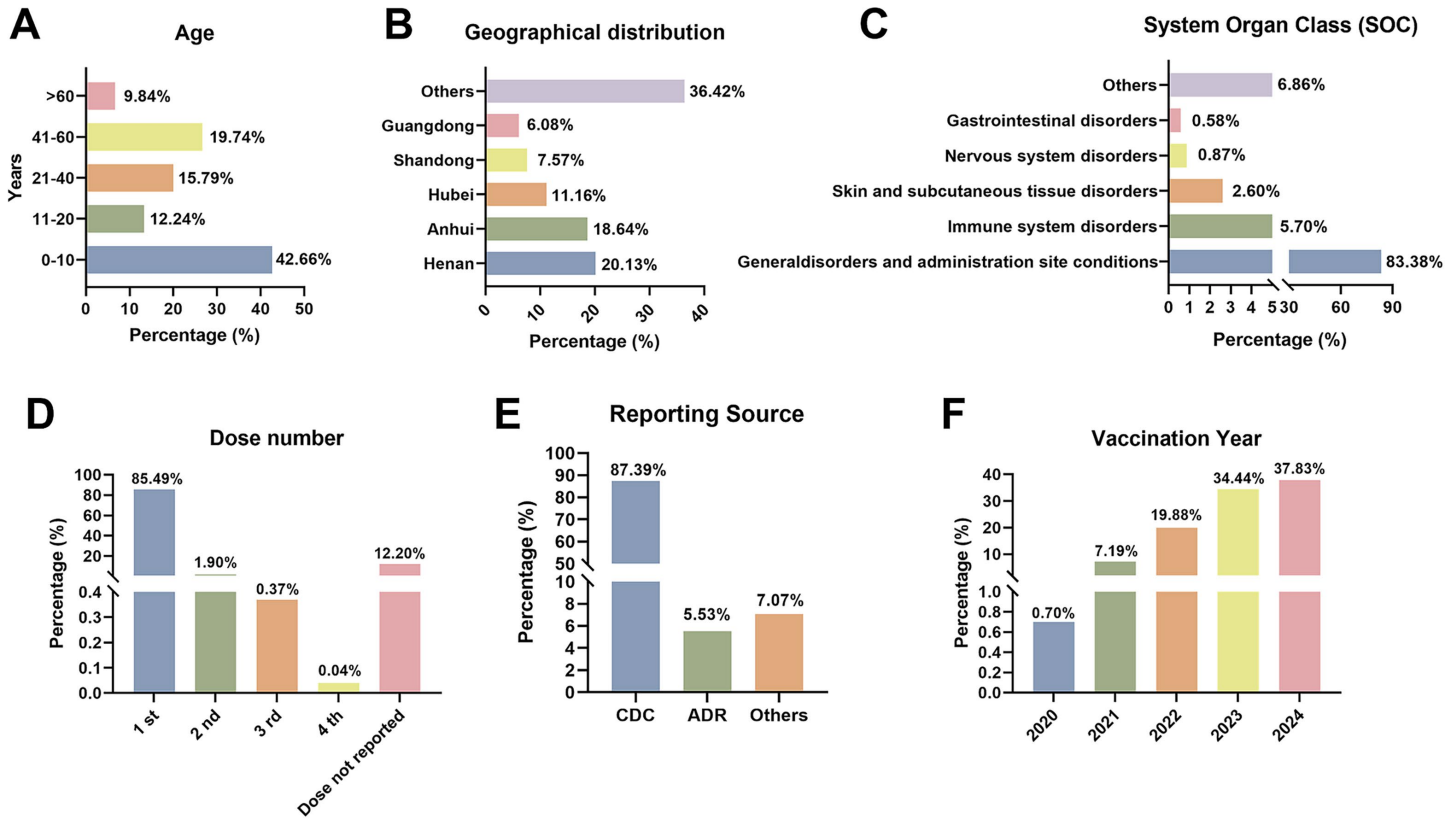
Distribution characteristics of adverse events following Yisheng Jun’an® rabies vaccine vaccination (2020–2024). **(A)** Age distribution of reported cases. **(B)** Geographical distribution of reported cases, with the top five provinces highlighted. **(C)** Classification of adverse events by System Organ Class (SOC), including general disorders, immune system disorders, and others. **(D)** Distribution of adverse events by vaccine dose (1st–4th dose, or dose not reported). **(E)** Sources of adverse event reports. **(F)** Distribution of reported cases by vaccination year (2020–2024).

Details are presented in Table 1. Among the reported cases, 1,127 were males (46.59%) and 1,292 were females (53.41%), with ages ranging from 0 to 97 years and a median age of 15 years (IQR: 6–45 years). Geographically, the majority of cases were concentrated in five provinces--Henan (487 cases, 20.13%), Anhui (451 cases, 18.64%), Hubei (270 cases, 11.16%), Shandong (183 cases, 7.57%), and Guangdong (147 cases, 6.08%)-together accounting for 63.58% of the total, while the remaining 881 cases (36.42%) were distributed across other provinces. In terms of System Organ Class (SOC), general disorders and administration site conditions were the most frequently reported (2017 cases, 83.38%), followed by immune system disorders (138 cases, 5.70%) such as urticaria and allergic reactions. A total of 331 adverse events included information on vaccination dose. Adverse events were most commonly observed after the 1st dose (2068 cases, 85.49%) and decreased progressively with subsequent doses, with only one case (0.04%) reported after the 4th dose. Major local adverse reactions (e.g., swelling, induration) were predominantly concentrated in the first two doses. Regarding reporting sources, CDC reports accounted for 87.39% (2,114/2,419) of all cases, followed by ADR reports (5.53%, 134/2,419) and other sources (7.07%, 171/2,419. With respect to vaccination years, the proportion of adverse reactions ranged from 0.66% in 2020 (16/2419) to 37.83% in 2024 (915/2,419) (Table 1; Figure 1).

**Table 1:** Demographic and clinical characteristics of reported adverse events following Yisheng Jun’an® rabies vaccine vaccination (2020–2024).

Characteristics	Number (*n*)	Proportion (%)
Gender		
Male	1,127	46.59
Female	1,292	53.41
Age range (years)	0–97	
Median age (IQR) (years)	15 (6–45)	
0–10	1,032	42.66
11–20	296	12.24
21–40	382	15.79
41–60	471	19.47
>60	238	9.84
Geographical distribution		
Henan	487	20.13
Anhui	451	18.64
Hubei	270	11.16
Shandong	183	7.57
Guangdong	147	6.08
Others	881	36.42
System organ class (SOC)		
General disorders and administration site conditions	2,017	83.38
Immune system disorders	138	5.70
Skin and subcutaneous tissue disorders	63	2.60
Nervous system disorders	21	0.87
Gastrointestinal disorders	14	0.58
Others	166	6.86
Dose number		
1st	2,068	85.49
2nd	46	1.90
3rd	9	0.37
4th	1	0.04
not reported	295	12.20
Reporting source		
CDC	2,114	87.39
ADR	134	5.53
Other	171	7.07
Vaccination year		
2020	16	0.70
2021	174	7.19
2022	481	19.88
2023	833	34.44
2024	915	37.83

### Clinical diagnosis of adverse reactions

As shown in Table 2, among local reactions, the most frequently reported were pruritus at the injection site (114 cases, 4.71%), macular erythema (46 cases, 1.90%), and induration (33 cases, 1.36%). Swelling (17 cases, 0.70%) and pain (21 cases, 0.87%) were less commonly observed. Systemic reactions were dominated by fever, which occurred in 1,639 cases (67.76%), followed by diarrhea (27 cases, 1.12%), arthralgia (23 cases, 0.95%), myalgia (10 cases, 0.41%), and other mild symptoms such as headache, vomiting, and dizziness.

**Table 2:** Reactogenicity and allergic reactions following rabies vaccine vaccination.

Type of reaction	Specific symptom	Number (*n*)	Proportion (%)
Local reactions	Swelling	17	0.70
	Macular erythema	46	1.90
	Induration	33	1.36
	Pain	21	0.87
	Pruritus (local)	114	4.71
	Others	152	6.28
Systemic reactions	Fever	1,639	67.76
	Headache	5	0.21
	Vomiting	7	0.29
	Dizziness	8	0.33
	Myalgia	10	0.41
	Arthralgia	23	0.95
	Diarrhea	27	1.12
	Others	124	5.13
Allergic reactions	Urticaria	22	0.91
	Allergic rash	99	4.09
	Anaphylaxis	7	0.29
	Angioedema	6	0.25
	Pruritus (systemic)	40	1.65
	Others	19	0.79
Total		2,419	100.00

Allergic reactions were relatively uncommon, with allergic rash being the most frequent (99 cases, 4.09%), followed by systemic pruritus (40 cases, 1.65%), urticaria (22 cases, 0.91%), and rare severe events including anaphylaxis (7 cases, 0.29%) and angioedema (6 cases, 0.25%). Overall, most adverse events were mild to moderate in nature, and serious allergic events were rare.

### Analysis of differences in adverse reactions among different subgroups

Details are shown in Table 3. No significant differences were observed in local reactions or systemic reactions between males and females (p > 0.05). Regarding age groups, systemic reactions had a higher incidence in the 0–10 years (37.99%, 919/2,419) and 21–40 years (12.08%, 292/2,419); Local reactions also had a higher incidence in the 0–10 years (4.67%, 113/2,419). Statistically significant differences in systemic and local reactions were observed across different age (p < 0.001).

**Table 3:** Differences in adverse reactions among different subgroups.

Subgroup	Category	Number (*n*) (%)	χ^2^	*p*
Gender				
local reactions	Male	181 (7.48%)	0.402	0.526
	Female	221 (9.14%)		
systemic reactions	Male	946 (39.11%)		
	Female	1,071 (44.27%)		
Age group (years)				
local reactions	0–10 years	113 (4.67%)	44.98	<0.001
	11–20 years	59 (2.44%)		
	21–40 years	90 (3.72%)		
	41–60 years	90 (3.72%)		
	>60 years	50 (2.07%)		
Systemic reactions	0–10 years	919 (37.99%)		
	11–20 years	237 (9.80%)		
	21–40 years	292 (12.076%)		
	41–60 years	381 (15.75%)		
	>60	188 (7.77%)		
Dose number				
local reactions	1st	305 (12.6%)	63.1681	<0.001
	2nd	18 (0.74%)		
	3rd	7 (0.29%)		
	4th	1 (0.04%)		
	Dose not reported	71 (2.94%)		
systemic reactions	1st	1763 (72.88%)		
	2nd	28 (1.16%)		
	3rd	2 (0.08%)		
	4th	0 (0%)		
	Dose not reported	224 (9.26%)		

Across different vaccine doses, the incidence of adverse reactions varied markedly. Local reactions were predominantly reported after the first dose (12.6%) and decreased sharply with subsequent doses (0.74% after the second, 0.29% after the third, and 0.04% after the fourth). Similarly, systemic reactions occurred primarily after the first dose (72.88%), while later doses showed substantially fewer reports (1.16% after the second, 0.08% after the third, and none after the fourth). There are statistically significant differences in the incidence rates of systemic and local reactions across different doses (p < 0.001).

## Discussion

This nationwide post-marketing surveillance study (2020–2024) assessed the safety profile of Yisheng Jun’an® freeze-dried human rabies vaccine (Vero cell). Based on 2,419 reported cases, the vaccine demonstrated an overall favorable safety profile, consistent with established patterns of inactivated rabies vaccines (12, 13). The overall incidence was 7.406 per 100,000 doses, with systemic reactions (83.38%) predominating over local reactions (16.62%). Most events were mild to moderate, and serious adverse events accounted for only 0.7% (17 cases) with no deaths, reinforcing a favorable benefit–risk balance.

By MedDRA classification, general disorders and administration site conditions were most common (83.38%), followed by immune system disorders (5.7%), such as urticaria (14). This distribution aligns with the immunological mechanism of vaccination, where immune activation may trigger transient systemic or allergic manifestations (15). Severe allergic events were extremely rare (anaphylaxis rate: 0.000021%), lower than the global rates reported for licensed rabies vaccines (14). A dose-dependent trend was also observed, with both local and systemic reactions most frequent after the first dose and decreasing markedly thereafter, reflecting the priming effect of initial antigen exposure (16, 17).

Age and gender differences were observed. Specifically, Children (0–10 years) exhibited the highest incidence of systemic reactions (37.99%), which may be attributed to immature immune regulatory mechanisms (18). Moreover, females reported a higher incidence of adverse events compared to males (53.41% vs. 46.59%), consistent with previous studies indicating stronger immune responses and gender-related reporting differences (19, 20). Geographical distribution was relatively balanced (top five provinces accounted for 65.58% of cases), while annual reports increased markedly in 2023–2024 (72.26% of cases), reflecting vaccine uptake and improved surveillance rather than a true rise in incidence (21). It is noteworthy that differences across regions in healthcare monitoring levels, clinician awareness of reporting, and public willingness to report may lead to reporting bias.

Compared with other Vero cell rabies vaccines, such as purified Vero cell rabies vaccine (PVRV), our data showed a higher proportion of systemic reactions (83.38%), with fever (67.76%) being the most common (10, 22, 23). This may reflect broader case definitions in post-marketing surveillance compared to pre-licensure trials. Importantly, the standard five-dose PEP schedule used in China was confirmed to be safe in this cohort. Alternative dose-sparing regimens warrant further investigation (24, 25).

Due to the absence of a control group in this post-marketing surveillance study, we performed indirect comparisons with published safety data from other Vero cell rabies vaccines to contextualize the observed adverse event incidence. A phase IV clinical trial on a rabies vaccine using the CTN-1 V strain and Vero cell platform the same production platform as YSJA reported that adverse reactions were mainly pyrexia and pain at the injection site, with severity mostly Grade 1 and concentrated within 0–3 days post-vaccination; no Grade 3 or above adverse events nor serious adverse events related to the vaccine were observed (26). This finding is consistent with our observation that the majority of adverse events were mild-to-moderate and transient. Another study on the inter-batch consistency of the CTN-1 V human rabies vaccine (Vero cell) reported an overall adverse event incidence of 54.4% under active clinical trial monitoring, with no significant differences in AE rates among three consecutive commercial-scale batches, demonstrating reliable batch-to-batch safety consistency (27). The much higher AE incidence reported in active clinical trial settings, compared with our passive surveillance incidence of 7.406 per 100,000 doses (0.0074%), reflects fundamental differences in surveillance methodology: active prospective monitoring with structured follow-up captures mild and transient reactions that are typically underreported in passive spontaneous reporting systems. Further supporting this interpretation, an active surveillance study in Jiangsu Province (2023–2024) using a mobile application for prospective follow-up reported overall adverse reaction rates of 2.10% for the 4-dose Zagreb regimen and 2.70% for the 5-dose Essen regimen among 2,000 participants (13). Another active post-marketing surveillance study in India on Rabivax-S, a Vero cell-based rabies vaccine, reported that among 991 vaccinees who received a five-dose intramuscular post-exposure prophylaxis regimen, a total of 69 adverse events were reported in 64 patients, of which 23 adverse events in patients were assessed as causally related to Rabivax-S (approximately 2.32%), while 26 adverse events were related to co-administered Rabishield rabies monoclonal antibody (28). In contrast, the passive surveillance system in the present study, covering approximately 32.66 million administered doses, yielded an overall incidence of 7.406 per 100,000 doses (0.0074%). This substantial discrepancy is expected and reflects the well-established difference in sensitivity between active and passive pharmacovigilance approaches.

Several limitations should be noted. The absence of a control group limits causal inference regarding vaccine-attributable risks and prevents determination of whether the observed events are vaccine-specific or non-specific. The retrospective design may limit completeness of data, and mild reactions in primary care settings may be underreported (29, 30). Batch-related safety variations were not assessed, and only short-term adverse events were analyzed, leaving long-term safety unknown. Future prospective, multi-center studies with extended follow-up and active comparator arms will be essential to provide a more comprehensive evaluation of vaccine safety.

## Conclusion

Yisheng Jun’an® freeze-dried human rabies vaccine demonstrates a favorable safety profile in real-world use, with low rates of serious adverse events and predictable reactogenicity patterns. The dose-dependent decline in adverse reactions, higher allergic rash incidence in younger populations, and predominance of mild systemic events align with global data on rabies vaccines. These findings support the continued use of this vaccine for post-exposure prophylaxis, emphasizing the importance of robust post-marketing surveillance to monitor long-term safety in diverse populations. Future efforts should focus on enhancing reporting completeness, particularly for mild reactions, and exploring risk factors (e.g., comorbidities) to better characterize vulnerable subgroups. Ultimately, maintaining high vaccination coverage remains critical for rabies prevention, as the disease is nearly 100% fatal without timely prophylaxis.

## Data Availability

The original contributions presented in the study are included in the article/supplementary material, further inquiries can be directed to the corresponding author/s.
